# A robust Bayesian bias‐adjusted random effects model for consideration of uncertainty about bias terms in evidence synthesis

**DOI:** 10.1002/sim.9422

**Published:** 2022-04-29

**Authors:** Ivette Raices Cruz, Matthias C. M. Troffaes, Johan Lindström, Ullrika Sahlin

**Affiliations:** ^1^ Centre for Environmental and Climate Science Lund University Lund Sweden; ^2^ Department of Biology Lund University Lund Sweden; ^3^ Department of Mathematical Sciences Durham University Durham United Kingdom; ^4^ Centre for Mathematical Sciences Lund University Lund Sweden

**Keywords:** imprecise probability, meta‐analysis, risk of bias, robust Bayesian analysis

## Abstract

Meta‐analysis is a statistical method used in evidence synthesis for combining, analyzing and summarizing studies that have the same target endpoint and aims to derive a pooled quantitative estimate using fixed and random effects models or network models. Differences among included studies depend on variations in target populations (ie, heterogeneity) and variations in study quality due to study design and execution (ie, bias). The risk of bias is usually assessed qualitatively using critical appraisal, and quantitative bias analysis can be used to evaluate the influence of bias on the quantity of interest. We propose a way to consider ignorance or ambiguity in how to quantify bias terms in a bias analysis by characterizing bias with imprecision (as bounds on probability) and use robust Bayesian analysis to estimate the overall effect. Robust Bayesian analysis is here seen as Bayesian updating performed over a set of coherent probability distributions, where the set emerges from a set of bias terms. We show how the set of bias terms can be specified based on judgments on the relative magnitude of biases (ie, low, unclear, and high risk of bias) in one or several domains of the Cochrane's risk of bias table. For illustration, we apply a robust Bayesian bias‐adjusted random effects model to an already published meta‐analysis on the effect of Rituximab for rheumatoid arthritis from the Cochrane Database of Systematic Reviews.

## 1 INTRODUCTION

Meta‐analysis is a statistical method to combine, analyze and summarize the results of studies that have the same target endpoint and calculate a quantitative estimate of the overall effect (pooled treatment/intervention effect) using fixed and random effects models^1^, ^2^ or network models.^3^, ^4^ There are several factors to consider in meta‐analysis such as heterogeneity and bias. Heterogeneity, arising from between study variations in populations, interventions, exposures and outcome measures, can be considered when specifying the meta‐analysis model. The potential of errors and biases (a.k.a. the risk of bias) due to differences in the design and conduct of the studies^1^, ^5^ is usually assessed qualitatively using critical appraisal.^2^, ^6^ The validity of the estimate of the overall effect in a meta‐analysis is affected by the quality of data, and uncertainty associated with the model (and parameters within the model). van der Bles et al^6^ conclude that these two levels of uncertainty, which they refer to as indirect uncertainty (ie, quality of the underlying knowledge eg, expressed by a reflexive summary of our confidence in the models or the experts) and direct uncertainty (ie, quantitative terms/expressions of uncertainty such as, a probability distribution or confidence interval) respectively, are usually communicated side by side. These two levels of uncertainty are both relevant, but can be both confusing and difficult to combine when making decisions. Therefore approaches to turn indirect uncertainty into direct uncertainty are useful.^6^

In practice, an analyst has the following alternatives to consider indirect uncertainty when characterizing direct uncertainty: (1) to remove studies with a high risk of bias and conduct the analysis with the best available evidence (ie, high quality studies), (2) to evaluate using sensitivity analysis the influence of including studies of lower quality in the meta‐analysis, or (3) to include all (or a selection of) studies, but adjust for bias.^7^ The last option can be carried out using quantitative bias analysis (or bias adjustment).^8^, ^9^, ^10^ Quantitative bias analysis is a method that requires the meta‐analysis model to be extended with bias adjustments (eg, additive or proportional adjustments of study specific errors in the model),^5^, ^11^, ^12^ and additional expert judgment^5^, ^6^, ^13^ on bias terms.

Bias terms in quantitative bias analysis are different from parameters. Parameters in statistical models are fixed quantities that we are uncertain about, but want to estimate using statistical inference.^6^ The bias terms are here treated as uncertain but fixed quantities that, in contrast to other parameters, we are not trying to learn. These bias terms are informed by expert judgment of detailed qualitative information on the design and execution of the studies. In practice, experts may be ignorant about bias terms or struggle to specify them by single values. Spiegelhalter and Best^11^ explored different choices of bias terms using sensitivity analysis. Turner et al^5^ elicited quantitative distributions for bias terms based on expert's judgments. Verde^12^ considered bias terms as scale random variables, modeled by a probability distribution. An alternative approach which avoids mixing uncertainties in bias terms and parameters, is to characterize ignorance or ambiguity about bias terms by a set of bias terms, and thus, it is not necessary to specify a unique value.

The aim of this article is to propose a way to consider uncertainty, arising from ambiguity or ignorance about bias terms, by modeling bias in a bias‐adjusted random effects model with imprecision, and use robust Bayesian analysis to estimate the overall effect. Robust Bayesian analysis is here seen as Bayesian updating performed over a set of coherent probability distributions, resulting in a set of posterior distributions for the quantity of interest. In this case, the set of posteriors are the result of the set of bias terms. Hence, the suggested robust Bayesian bias analysis characterizes uncertainty in the overall effect by bounded probability,^14^, ^15^, ^16^ where the differences between bounds (ie, the degree of imprecision) in the overall effect is a result of ambiguity or ignorance about the bias terms. In this way, it is possible to evaluate the impact of bias and the impact of uncertainty associated with the bias separately.

We use a bias‐adjusted random effects model with an additive bias (the study specific treatment effect is modeled as the sum of an overall effect, a study specific random effect and a study specific error) associated with each study specific effect (as was done in Turner et al^5^ and Spiegelhalter and Best^11^). We propose a way to specify the set of bias terms for all studies by considering information from the Cochrane risk of bias table (RoB table). For illustration, we apply a robust Bayesian bias‐adjusted random effects model to a published meta‐analysis about the effect of Rituximab for rheumatoid arthritis from the Cochrane Database of Systematic Reviews.^17^

In what follows, we first describe the robust Bayesian bias‐adjusted random effects model (Section 2). Next, we propose how to specify a set of coherent bias terms (in the model referred to as study qualities) using qualitative judgments about study quality from the RoB table (Section 3). We then present an application of robust Bayesian quantitative bias analysis to a systematic review about the effect of Rituximab (Section 4). We conclude with a discussion (Section 5).

## 2 MODEL SPECIFICATION

### 2.1 A random effects model

Consider a meta‐analysis for binary outcome of K studies where $N_{ij}$ denotes the total number of patients in group j (j=1 Control and j=2 Treatment) in study i (i=1,…,K) and $r_{ij}$ denotes the number of patients who have had a positive response respectively.

The number of patients $r_{ij}$ can be modeled with a binomial distribution as follows:

(1) $$r_{ij}|p_{ij}∼\text{Binomial}(p_{ij},N_{ij}).$$

The probability of success $p_{ij}$ can be transformed using a link function (eg, logit) as follows:

(2) $$\begin{array}{ll} \text{logit}(p_{i1}) & =\ln\left(\frac{p_{i1}}{1-p_{i1}}\right)=\beta_{i}, \end{array}$$

(3) $$\begin{array}{ll} \text{logit}(p_{i2}) & =\ln\left(\frac{p_{i2}}{1-p_{i2}}\right)=\beta_{i}+\delta_{i}, \end{array}$$

where $\beta_{i}$ is the log‐odds ratio (“log‐OR”) for the control group and $\delta_{i}$ is the specific treatment effect on the “log‐OR” scale in study i. The specific treatment effect being evaluated in the ith study, $\delta_{i}$, can be expressed as the sum of the overall effect, μ, the study specific random effect, $\theta_{i}$ and study specific error, $\phi_{i}$:

(4) $$\delta_{i}=\mu +\theta_{i}+\phi_{i},$$

on the “log‐OR” scale.

In addition, uncertainty about $\theta_{i}$ and $\phi_{i}$ can be described by

(5) $$\begin{array}{ll} \theta_{i}|\sigma_{\theta }^{2} & ∼\text{Normal}(0,\sigma_{\theta }^{2}), \end{array}$$

(6) $$\begin{array}{ll} \phi_{i} & ∼\text{Normal}(0,\sigma_{\phi_{i}}^{2}), \end{array}$$

where $\sigma_{\theta }^{2}$ represents between study variability and $\sigma_{\phi_{i}}^{2}$ is the variance of the bias (study specific error). Here, Equation (6) represents uncertainty in a single trial of a study. In repeated trials, which we are not modeling here, there may be correlation between repeated trials of the same study. We assume that the expectation of study specific random effect and study specific error, $\theta_{i}$ and $\phi_{i}$, is 0 because we do not consider that a random effect or bias would favor either the treatment or the control group. Then, uncertainty about $\delta_{i}$ can be represented by

(7) $$\delta_{i}|\mu ,\sigma_{\theta }^{2}∼\text{Normal}(\mu ,\sigma_{\theta }^{2}+\sigma_{\phi_{i}}^{2}).$$

### 2.2 Bias adjustment model

To adjust for bias, Equation (7) is expressed as

(8) $$\delta_{i}|\mu ,\sigma_{\theta }^{2}∼\text{Normal}\left(\mu ,\frac{\sigma_{\theta }^{2}}{q_{i}}\right),$$

where $q_{i}=\frac{\sigma_{\theta }^{2}}{\sigma_{\theta }^{2}+\sigma_{\phi_{i}}^{2}}$ is a bias term that can be interpreted as the quality of study i ^5^, ^11^ (therefore, in what follows, bias terms are referred to as study qualities). Study quality represents how large a proportion of total variance is due to between study variability compared to bias/design uncertainty. The case $q_{i}=1$ represents the situation where there is no bias whereas $q_{i}=0.5$ implies equal between study variability and variance of the bias term for study i. For example, a high quality study is expected to have a small error in relation to the between study variability and should therefore have a relatively high study quality, for example, q=0.95 (or other value close to 1). A low quality study should accordingly have a low quality, for example, q=0.1 (or other value close, but strictly larger than zero). The study qualities influence the relative contribution of each study to the likelihood of the model and hence the estimation of the overall effect.

### 2.3 Bayesian inference

**FIGURE 1:**
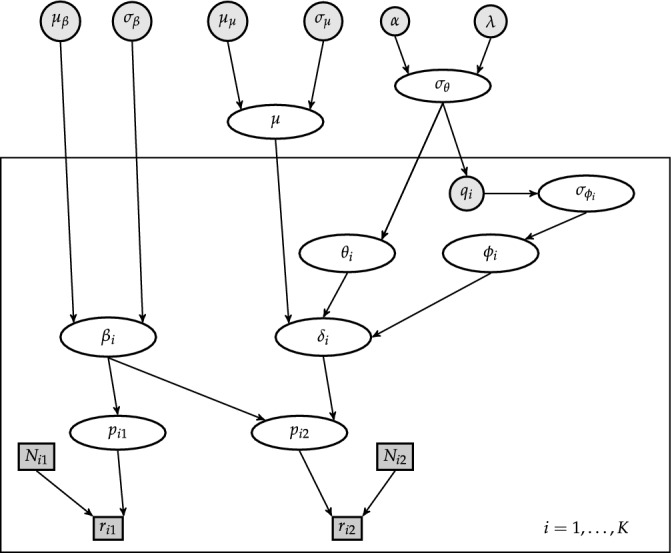
A probabilistic graphical representation of the Bayesian bias‐adjusted random effects model. Unknown quantities (parameters) are represented by white ellipses, for which priors are specified with fixed hyperparameters (gray circles). Observations (gray squares) are coming from K studies (the plate). The bias terms are fixed and therefore denoted by a gray circle

To implement the model in a Bayesian framework, the following prior distributions are specified for the parameters

(9a) $$\begin{array}{ll} \beta_{i} & ∼\text{Normal}(\mu_{\beta },\sigma_{\beta }^{2}), \end{array}$$

(9b) $$\begin{array}{ll} \mu & ∼\text{Normal}(\mu_{\mu },\sigma_{\mu }^{2}), \end{array}$$

(9c) $$\begin{array}{ll} \sigma_{\theta }^{2} & ∼\text{InvGamma}(\alpha ,\lambda ), \end{array}$$

where $\mu_{\beta }$, $\sigma_{\beta }^{2}$, $\mu_{\mu }$, $\sigma_{\mu }^{2}$, α, and λ are hyperparameters for the normal and inverse gamma prior distributions. The study qualities $q_{i}$ can be interpreted as hyperparmeters for the bias adjustment part, or as auxiliary parameters as they are not integrated out in the analysis. The Bayesian bias‐adjusted random effects model is a probabilistic graphical model (Figure 1) that represents probabilistic dependencies among data, parameters and hyperparameters.

### 2.4 Robust Bayesian inference

The Bayesian bias‐adjusted random effects model is extended to a robust Bayesian framework by keeping the previously specified prior distributions and specifying a set of study qualities ℳ resulting in:

(10) $$\delta_{i}|\mu ,\sigma_{\theta }^{2}∼\text{Normal}\left(\mu ,\frac{\sigma_{\theta }^{2}}{q_{i}}\right)\;\text{where}\;q_{i}\in ℳ.$$

The robust Bayesian bias‐adjusted random effects model allows us to evaluate the impact of uncertainty about bias terms, $q_{i}$, on uncertainty of the quantity of interest (eg, the overall effect of the intervention, μ). This means that for different values $q_{i}\in ℳ$, there are different distributions for $\delta_{i}$ and thus, specifying a set of study qualities results in bounds on the probabilities that characterize uncertainty in the quantity of interest. The difference between lower and upper bounds for the probabilities expressing uncertainty in the quantity of interest is therefore a result of ambiguity or ignorance about the bias terms in the quantitative bias analysis.

Uncertainty about the overall effect, μ, is summarized by bounds on the expected overall effect, bounds on the 5th percentile, and bounds on P(μ>t), the probability of the overall effect, μ, exceeding a decision relevant threshold, t∈ℝ. For instance, the posterior expected overall effect $E_{q}(\mu )$ is calculated by a multiple integral of μ times the posterior distribution over the domains of the parameters $\beta_{1},\beta_{2},\beta_{3},\beta_{4},\delta_{1},\delta_{2},\delta_{3},\delta_{4},\mu ,\sigma_{\theta }^{2}$ (see Appendix A for details). Then, lower and upper bounds on the expected overall effect are given by

(11) $$\begin{array}{ll} {\underline{E}}_{q}(\mu ) & =\underset{q\in ℳ}{\inf}E_{q}(\mu ), \end{array}$$

(12) $$\begin{array}{ll} {\bar{E}}_{q}(\mu ) & =\underset{q\in ℳ}{\sup}E_{q}(\mu ). \end{array}$$

Bounds are here estimated using a discretization of the elements of the set of study qualities, in particular, we specify a finite set of study quality using a grid (details are given in Section 4). Then, for each study quality in this set, we estimate the expectation, percentile and exceedance probability of the overall effect using Markov chain Monte Carlo (MCMC) sampling which draws samples from the posterior distribution. Finally, the lower and upper bounds are approximated by the minimum and the maximum values of each estimated quantity.

## 3 QUANTIFICATION OF STUDY QUALITY FROM THE COCHRANE'S RISK OF BIAS TABLE

We describe a way to incorporate qualitative judgments about risk of bias in a bias‐adjusted meta‐analysis. In particular, we show how to transform qualitative judgments about risk of bias into quantitative expressions (ie, a coherent set of study qualities) and thereby characterizing uncertainty in the specification of bias (study quality) by bounded probability.

The Cochrane's risk of bias table (RoB table) is the recommended tool for assessing the risk of bias in each included study in Cochrane reviews.^18^ A RoB table takes into account the following domains: random sequence generation (selection bias), allocation concealment (selection bias), blinding of participants and personnel (performance bias), incomplete outcome data (attrition bias), selective outcome reporting (reporting bias) and other potential sources of bias.^18^ Each domain is assessed individually and classified in three categories: low, unclear and high risk of bias.^18^ Here, we describe a way to use this information that only requires a specification of the bounds for the lowest and highest value on the bias term $q_{i}$ across all studies i.

### 3.1 Considerations for rating studies

Let us consider a single domain of the RoB table for establishing a rating between the studies. Let $q_{\left(+\right)}$, $q_{\left(?\right)}$ and $q_{\left(-\right)}$ denote the study quality of studies with low, unclear and high risk of bias, respectively. The approach is explained using a hypothetical systematic review with 6 studies ($S_{1},\ldots ,S_{6}$) which are classified as follows: $S_{1}$ and $S_{2}$ with a low risk of bias, $S_{3}$ and $S_{4}$ with an unclear risk of bias and $S_{5}$ and $S_{6}$ with a high risk of bias. The set of quantitative bias terms are constructed as follows:

1. Group the studies according to their risk of bias: low (+), unclear (?) and high (−).
2. All studies belonging to a group with a clear risk of bias are assumed to have the same study quality. For example: $q_{1}=q_{2}=q_{\left(+\right)}$ and $q_{5}=q_{6}=q_{\left(-\right)}$.
3. The quality of studies belonging to the group with an unclear risk of bias are not necessarily equal and are somewhere in between $q_{\left(-\right)}\leq q_{\left(?\right)}\leq q_{\left(+\right)}$. For example: $q_{\left(-\right)}\leq q_{3}\leq q_{\left(+\right)}$ and $q_{\left(-\right)}\leq q_{4}\leq q_{\left(+\right)}$.
4. Assign a range representing the lower and upper bounds for the bias terms in the low and high risk of bias categories.

In what follows, we let $q_{\left(+\right)}$ take values between 0.5 and 0.95, which corresponds to situations where between study variability contributes more to the total variance compared to the variance of study specific errors. We let $q_{\left(-\right)}$ take values between 0.1 and 0.5, which corresponds to situations where the variance of study specific errors contributes more than between study variability to the total variance. For the group of studies with unclear risk of bias, we use the most extreme bounds for the high and low risk of bias group, that is, $0.1=\underline{q_{\left(-\right)}}\leq q_{\left(?\right)}\leq \bar{q_{\left(+\right)}}=0.95$.

## 4 APPLICATION

### 4.1 Data

Data are taken from a systematic review about Rituximab for rheumatoid arthritis from the Cochrane Database of Systematic Reviews. For this example, we include four studies investigating the effect of Rituximab plus metrotexato (RT + MTX) vs placebo plus metrotexato (MTX) in patients with rheumatoid arthritis. The effect is assessed by the number of patients who have improved by at least 50% on the American College of Rheumatology scale (ACR50) at week 24.^17^ The outcomes of the studies and their assessment of risk of bias are shown in Tables 1 and 2, see Reference 17(p. 15), respectively.

**TABLE 1:** Summary of studies^17^

	Control group (MTX)		Treatment group (RT + MTX)	
Study name	Response	Total	Response	Total
REFLEX (Study 1)	10	201	80	298
WA16291 (Study 2)	5	40	17	40
DANCER (Study 3)	16	122	41	122
SERENE (Study 4)	16	172	44	170
Total	47	535	182	630

**TABLE 2:** Risk of bias table taken from the systematic review^17^

Study name	1	2	3	4	5	6
REFLEX (Study 1)	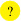	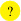	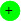	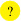	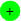	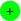
WA16291 (Study 2)	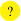	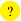	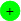	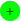	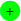	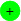
DANCER (Study 3)	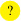	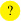	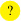	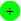	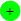	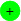
SERENE (Study 4)	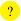	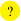	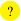	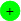	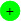	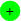

**1**, Random sequence generation (selection bias).

**2**, Allocation concealment (selection bias).

**3**, Blinding of participants and personnel (performance bias).

**4**, Incomplete outcome data (attrition bias).

**5**, Selective outcome reporting (reporting bias).

**6**, Other potential sources of bias.

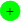
, Low risk of bias.

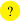
, Unclear risk of bias.

### 4.2 Prior specification

Noninformative prior distributions are used on the parameters $\beta_{i},\mu$, and $\sigma_{\theta }^{2}$ which follow normal and inverse gamma distributions, Equation (9). The following hyperparameter values are assigned: $\mu_{\beta }=0$, $\sigma_{\beta }=10$, $\mu_{\mu }=0$, $\sigma_{\mu }=10$, α=0.01, and λ=0.01. Let us note that the estimated overall effect of the Bayesian bias‐unadjusted random effects model (Table 3) using these hyperparameter values gives estimates similar to the one, in the published Cochrane meta‐analysis.^17^

**TABLE 3:** Bounds on expected value, exceedance probability and 5% percentile of the overall effect, μ, comparing Rituximab plus metrotexato (treatment) against placebo plus metrotexato (control) for robust bias adjusted meta‐analysis considering different groups of bias domains

Bias domain	Quantity of interest	Lower bound	$q_{*}$	Upper bound	$q^{*}$
**1, 2**	E(μ)	1.328	(0.10, 0.10, 0.76, 0.95)	1.646	(0.95, 0.86, 0.10, 0.10)
P(μ>1)	0.886	(0.10, 0.10, 0.76, 0.76)	0.983	(0.95, 0.86, 0.10, 0.10)
$P_{5\%}$	0.826	(0.10, 0.10, 0.19, 0.19)	1.169	(0.95, 0.86, 0.10, 0.10)
**3**	E(μ)	1.461	(0.86, 0.86, 0.86, 0.78)	1.634	(0.86, 0.86, 0.10, 0.10)
P(μ>1)	0.945	(0.50, 0.50, 0.42, 0.50)	0.982	(0.95, 0.95, 0.10, 0.10)
$P_{5\%}$	0.982	(0.59, 0.59, 0.51, 0.59)	1.159	(0.95, 0.95, 0.10, 0.10)
**4**	E(μ)	1.350	(0.10, 0.91, 0.91, 0.91)	1.476	(0.59, 0.59, 0.59, 0.59)
P(μ>1)	0.902	(0.10, 0.95, 0.95, 0.95)	0.956	(0.86, 0.86, 0.86, 0.86)
$P_{5\%}$	0.881	(0.10, 0.59, 0.59, 0.59)	1.025	(0.82, 0.86, 0.86, 0.86)
**5, 6**	E(μ)	1.462	(0.50, 0.50, 0.50, 0.50)	1.478	(0.90, 0.90, 0.90, 0.90)
P(μ>1)	0.945	(0.50, 0.50, 0.50, 0.50)	0.955	(0.85, 0.85, 0.85, 0.85)
$P_{5\%}$	0.982	(0.50, 0.50, 0.50, 0.50)	1.020	(0.85, 0.85, 0.85, 0.85)
All **1, 2, 3 , 4, 5, 6**	E(μ)	1.356	(0.10, 0.95, 0.95, 0.95)	1.638	(0.87, 0.95, 0.10, 0.10)
P(μ>1)	0.905	(0.10, 0.36, 0.36, 0.36)	0.982	(0.87, 0.87, 0.10, 0.10)
$P_{5\%}$	0.847	(0.10, 0.10, 0.10, 0.10)	1.161	(0.87, 0.87, 0.10, 0.10)
Bias‐unadjusted model	E(μ)	1.471	‐	1.471	‐
P(μ>1)	0.998		0.998	
$P_{5\%}$	1.029		1.029	

*Note*: $q_{*}$ and $q^{*}$ are the values for the bias terms where the bounds are obtained. The probability of the overall effect exceeding a threshold t (where t=1 for illustrative purposes).

**1**, Random sequence generation (selection bias).

**2**, Allocation concealment (selection bias).

**3**, Blinding of participants and personnel (performance bias).

**4**, Incomplete outcome data (attrition bias).

**5**, Selective outcome reporting (reporting bias).

**6**, Other potential sources of bias.

### 4.3 Bias adjustment based on study quality

Bias was accounted for by rating the studies according to each bias domain in the RoB table of the systematic review separately. For example, the effect of selection bias due to random sequence generation was evaluated separately from attrition bias due to incomplete outcome data. We illustrate each possible case according to each different domain listed in Table 2. Specifically, we consider four cases corresponding to each of the four distinct columns in the table and an additional case corresponding to multiple domains.

- **Bias domains 1 and 2**
  The impact of selection biases due to random sequence generation and allocation concealment domains is similar because all the studies are rated with an unclear risk of bias. Following the considerations in Section 3.1:
  ‐ Studies with unclear risk of bias can have different study qualities and satisfy
(13) $$0.1\leq q_{i}\leq 0.95,\;i=1,\ldots ,4$$
  which yields to the following set
(14) $${𝒮}_{1}={𝒮}_{2}=\left\{\begin{array}{c} q:=(q_{1},q_{2},q_{3},q_{4}):\\ 0.1\leq q_{i}\leq 0.95,\;i=1,\ldots ,4 \end{array}\right\}.$$
  A regular grid of 10×10×10×10 of study quality q is used for estimating the bounds, which results in 10 000 study quality values, q. More specifically, we considered $q_{i}$ equally spaced between 0.10 and 0.95.
- **Bias domain 3**
  Based on the impact of performance bias due to blinding of participants and personnel domain, the studies are grouped as follows: REFLEX (Study 1) and WA16291 (Study 2) in the category of low risk of bias and DANCER (Study 3) and SERENE (Study 4) in the category of unclear risk of bias. Then, we get:
  ‐ Studies with low risk of bias have equal study qualities and studies with unclear risk of bias can have different study qualities and satisfy
(15) $$q_{3}\leq q_{1},\;\text{and}\;q_{4}\leq q_{1},\;\text{where}\;q_{1}=q_{2}.$$
  Here, we make use of the previously established bounds for studies with low risk of bias and in the absence of studies with high risk of bias, 0.1 is then used as the lower bound for studies with unclear risk of bias. The resulting set is
(16) $${𝒮}_{3}=\left\{\begin{array}{c} q:=(q_{1},q_{2},q_{3},q_{4}):\\ q_{2}=q_{1},\\ 0.1\leq q_{3}\leq q_{1},\\ 0.1\leq q_{4}\leq q_{1},\\ 0.5\leq q_{1}\leq 0.95 \end{array}\right\}.$$
  For estimating the bounds, we use a discretization of the convex combination of the extreme points of this set which can be seen as the weighted sum of the extreme points of the set. In details, these are the extreme points of ${𝒮}_{3}$:
$$\begin{array}{ll} v_{1} & =(0.50,0.50,0.10,0.10),\;\\ v_{2} & =(0.95,0.95,0.10,0.10),\;\\ v_{3} & =(0.50,0.50,0.10,0.50),\;\\ v_{4} & =(0.95,0.95,0.10,0.95),\;\\ v_{5} & =(0.50,0.50,0.50,0.10),\;\\ v_{6} & =(0.95,0.95,0.95,0.10),\;\\ v_{7} & =(0.50,0.50,0.50,0.50),\;\\ v_{8} & =(0.95,0.95,0.95,0.95),\; \end{array}$$
  and study qualities are specified as
(17) $$q=\overset{8}{\underset{i=1}{\sum }}\alpha_{i}\cdot v_{i}\;\text{where}\;\overset{8}{\underset{i=1}{\sum }}\alpha_{i}=1\;\text{and}\;\alpha_{i}\geq 0.$$
  In Equation 17, $\alpha_{i}$ represents the weights. We used a grid spacing of 0.1 for each $\alpha_{i}$ (i=1,…,7) for estimating the bounds. Specifically, the following values are used:
$$\begin{array}{ll} \alpha_{1} & \in \{0,\;0.1,\ldots ,\;1\},\;\\ \alpha_{k} & \in \left\{0,\ldots ,1-\overset{k-1}{\underset{i=1}{\sum }}\alpha_{i}\right\},\;k=2,\ldots ,7\;\\ \alpha_{8} & =1-\overset{7}{\underset{i=1}{\sum }}\alpha_{i}.\; \end{array}$$
  Duplicate values of q are removed, which results in 736 study quality values, q.
- **Bias domain 4**
  Based on attrition bias due to incomplete outcome data, the studies are grouped as follows: WA16291 (Study 2), DANCER (Study 3) and SERENE (Study 4) in the category of low risk of bias and REFLEX (Study 1) in the category of unclear risk of bias. Then, we get:
  ‐ Studies with unclear risk of bias can have different study qualities and satisfy
(18) $$q_{1}\leq q_{2},\;\text{where}\;q_{2}=q_{3}=q_{4},$$
  similarly, we get
(19) $${𝒮}_{4}=\left\{\begin{array}{c} q:=(q_{1},q_{2},q_{3},q_{4}):\\ q_{2}=q_{3}=q_{4},\\ 0.1\leq q_{1}\leq q_{2},\\ 0.5\leq q_{2}\leq 0.95 \end{array}\right\}.$$
  Following the same procedure as in domain 3, the extreme points of ${𝒮}_{4}$ are:
$$\begin{array}{ll} v_{1} & =(0.10,0.95,0.95,0.95),\;\\ v_{2} & =(0.10,0.50,0.50,0.50),\;\\ v_{3} & =(0.50,0.50,0.50,0.50),\;\\ v_{4} & =(0.95,0.95,0.95,0.95).\; \end{array}$$
  Using a grid spacing of 0.1 of $\alpha_{i}$ (weights) we used the following values
$$\begin{array}{ll} \alpha_{1} & \in \{0,\;0.1,\ldots ,\;1\},\;\\ \alpha_{k} & \in \left\{0,\ldots ,1-\overset{k-1}{\underset{i=1}{\sum }}\alpha_{i}\right\},\;k=2,3,\;\\ \alpha_{4} & =1-\overset{3}{\underset{i=1}{\sum }}\alpha_{i}.\; \end{array}$$
  The weighted sum of the extreme points of this set yields 286 study quality values, q.
- **Bias domains 5 and 6**
  The impact of bias due to selective outcome reporting and other potential sources of bias domains are similar because all the studies are rated with a low risk of bias. Thus:
  ‐ All the studies in the group of low risk of bias have the same study quality
(20) $$q_{1}=q_{2}=q_{3}=q_{4},$$
  and therefore
(21) $${𝒮}_{5}={𝒮}_{6}=\left\{\begin{array}{c} q:=(q_{1},q_{2},q_{3},q_{4}):\\ q_{1}=q_{2}=q_{3}=q_{4},\\ 0.5\leq q_{1}\leq 0.95 \end{array}\right\}.$$
  In this case, 10 equally spaced values between 0.5 and 0.95 of $q_{1}$ are considered, which results in 10 study quality values, q.
- **Multiple bias domains**
  So far, we have focused on single domains of the RoB table. However, the proposed methodology could be extended or adapted, by making use of more than one domain of the risk of bias table. This requires a clear and transparent guidance on how to rate studies considering quality of data from several domains at once, as well as on how to prioritize domains.
  An example on how multiple bias domains could be combined for rating the studies is presented. The following considerations are taken into account:
  ‐ WA16291 (Study 2) should be better than the rest of the studies, (it has four domains with a low risk of bias and two with an unclear risk of bias),
  ‐ DANCER (Study 3) and SERENE (Study 4) have the same type of biases,
  ‐ It is not possible to relate REFLEX (Study 1) to DANCER (Study 3) or SERENE (Study 4) (we do not know if domain 3 and 4 are equally bad or if one is better than the other).
  which results in:
(22) $$q_{1}\leq q_{2},\;q_{3}\leq q_{2},\;\text{where}\;q_{3}=q_{4}.$$
  In this case, it is not possible to arrive to a final category of risk of bias without introducing a scoring rule (since all studies have domains with low and unclear risk of bias). Therefore, once again, we specify the most extreme bounds to account for uncertainty, in the specification of bias (study quality), resulting in:
(23) $${𝒮}_{\text{all}}=\left\{\begin{array}{c} q:=(q_{1},q_{2},q_{3},q_{4}):\\ q_{3}=q_{4},\\ 0.1\leq q_{1}\leq q_{2},\\ 0.1\leq q_{3}\leq q_{2},\\ 0.1\leq q_{2}\leq 0.95 \end{array}\right\}.$$
  The same procedure as in domains 3 and 4 is followed. The extreme points of ${𝒮}_{\text{all}}$ are:
$$\begin{array}{ll} v_{1} & =(0.10,0.10,0.10,0.10),\;\\ v_{2} & =(0.10,0.95,0.10,0.10),\;\\ v_{3} & =(0.10,0.95,0.95,0.95),\;\\ v_{4} & =(0.95,0.95,0.10,0.10),\;\\ v_{5} & =(0.95,0.95,0.95,0.95).\; \end{array}$$
  We use a grid spacing of 0.1 of $\alpha_{i}$, specifically,
$$\begin{array}{ll} \alpha_{1} & \in \{0,\;0.1,\ldots ,\;1\},\;\\ \alpha_{k} & \in \left\{0,\ldots ,1-\overset{k-1}{\underset{i=1}{\sum }}\alpha_{i}\right\},\;k=2,\ldots ,4,\;\\ \alpha_{5} & =1-\overset{4}{\underset{i=1}{\sum }}\alpha_{i},\; \end{array}$$
  which results in 839 study quality values, q.

### 4.4 Estimation of treatment effect adjusted for bias

**FIGURE 2:**
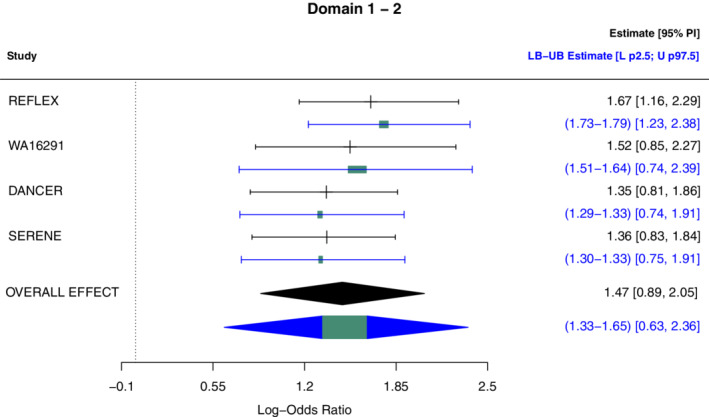
Forestplot of a meta‐analysis of the effectiveness of Rituximab plus metrotexato modified to show bounds on quantities of interest. Unadjusted and robust Bayesian bias‐adjusted random effects log‐odds ratios (with 95% PI) are displayed: (black) unadjusted model; (blue) robust bias‐adjusted random effects model. For the robust bias‐adjusted random effects model, bounds on the expected overall effect, the lower 2.5th percentile and the upper 97.5th percentile are shown

**FIGURE B1:**
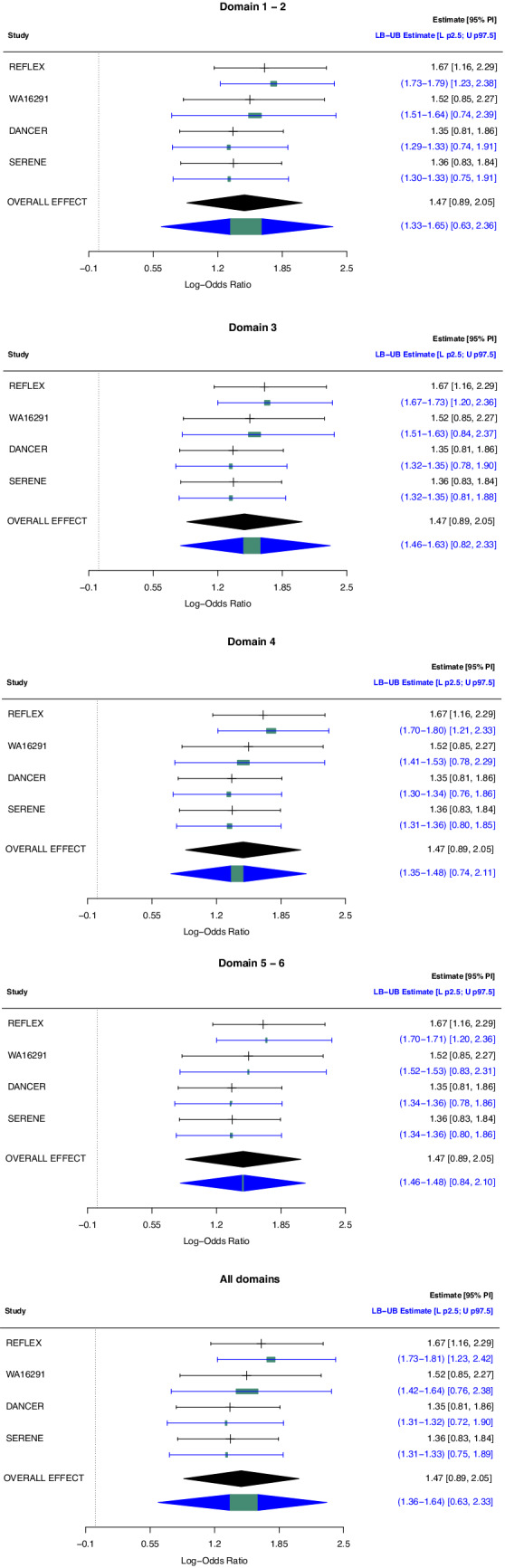
Forestplot of a meta‐analysis of the effectiveness of Rituximab plus metrotexato modified to show bounds on quantities of interest. Unadjusted and robust Bayesian bias‐adjusted random effects log‐odds ratios (with 95% PI) are displayed: (black) unadjusted model; (blue) robust bias‐adjusted random effects model. For the robust bias‐adjusted random effects model, bounds on the expected overall effect, the lower 2.5th percentile and the upper 97.5th percentile are shown

Study specific treatment effects and overall effect are estimated using a robust Bayesian bias‐adjusted random effects model. The model is implemented using MCMC sampling in JAGS through the *rjags* and *runjags* packages, which are R interfaces to JAGS (see https://github.com/Iraices/Robust_bias_adjustment for the code). Forestplots (a common graphical representation in meta‐analysis) are used to present the estimated overall effect and specific study effect using the *metafor* package for R. To express the results from robust Bayesian analysis, the forestplots from *metafor* were expanded with bounds on the expected overall effect, E(μ), the lower 2.5th percentile and the upper 97.5th percentile (Figure 2). The results of the random effects model with and without robust Bayesian bias‐adjustment are displayed for each risk of bias domain in Figure B1.

**FIGURE 3:**
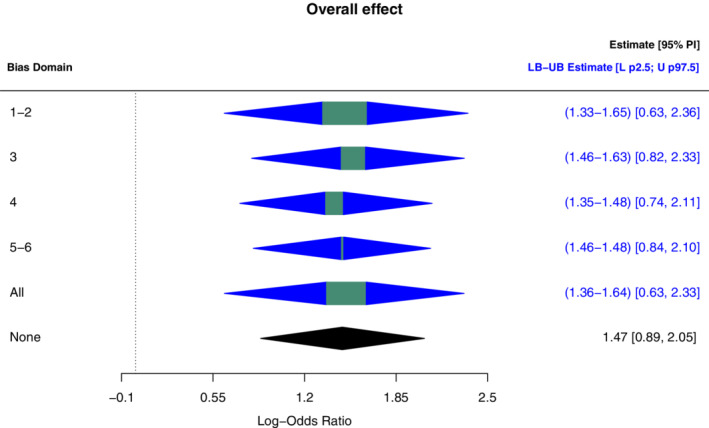
Uncertainty in the overall effect per bias domain. For the robust bias‐adjusted random effects model, lower and upper bounds on the expected overall effect, a lower bound on the 2.5th percentile and an upper bound on the 97.5th percentile are shown

Adjusting for bias associated with bias domains 1 and 2 changed the estimated overall effect from 1.47 to between 1.33 and 1.65; and resulted in a lower bound of the two sided 95% probability interval (ie, lower 2.5th percentile) to change from 0.89 to 0.63 (Figure 2). The difference between the lower and upper bound (ie, the degree of imprecision) of the expected overall effect varied when adjusting for different risk of bias domains (Figure 3). As expected, there is more imprecision in the estimated overall effect, when all studies have an unclear risk of bias (domain 1‐2). For bias domains 5 and 6, all studies have a low risk of bias and were, according to the considerations set up for constructing the study quality sets, given equal bias terms. As a consequence, the between study variability has a very high influence on the total variance compared to the variance of bias, and therefore the overall effect is very similar to the unadjusted model. For domains 3 and 4, all studies, except WA16291 (Study 2) swapped their risk of bias category, between low and unclear, respectively. This explains why the overall effect is adjusted upwards (domain 3) and downwards (domain 4) compared to the unadjusted case.

For a given domain, the values where the bounds are obtained, $q_{*}$ and $q^{*}$, can be different depending on the quantity of interest (Table 3). For domains 1 and 2, the lower bound for the expected overall effect is obtained at $q_{*}=(0.10,0.10,0.76,0.95)$ whereas the lower bound for the probability of the overall effect exceeding 1 is obtained at $q_{*}=(0.10,0.10,0.76,0.76)$. The values of $q_{*}$ and $q^{*}$ are not necessarily on the extreme points of the set. Hence, it may be difficult to know in advance the values of q where the bounds are obtained.

Adjusting for bias may reveal important aspects to consider when framing a conclusion in evidence synthesis. Here, the evidence in favor of Rituximab plus metrotexato (treatment group) from the published meta‐analysis^17^ remains strong after adjusting for bias. We can see that the lower 2.5th percentile of the overall effect do not cross the reference value zero, for all risk of bias domains (Figure 3). Thus, the conclusion is robust to uncertainty (including ambiguity of the specification of bias terms).

## 5 DISCUSSION AND CONCLUSION

Quantitative bias analysis^8^, ^9^ is a statistical approach to combine direct and indirect uncertainty in evidence synthesis. Indirect uncertainty is often given as qualitative judgments on risk of biases.^6^ Hence, bias analysis requires qualitative judgments of risk of bias to be transformed into quantitative expressions of uncertainty associated with estimates from studies (bias terms). In practical applications, it can be challenging to come up with precise bias terms. In this article, we therefore propose robust Bayesian bias analysis as a way to consider ambiguity or ignorance about how to quantify bias terms in meta‐analysis, that distinguishes the impact of uncertainty about the bias terms from other uncertainties in the model. The proposed approach is a novel way to, in a structured way, use qualitative information concerning risk of biases in quantitative bias analysis, and bridge the gap between qualitative and quantitative expressions of uncertainty. This is done by characterizing uncertainty about the bias terms by a set of bias terms, whereas parameter uncertainty is expressed by subjective probability. These two types of uncertainty are sometimes referred as second and first order uncertainty respectively.^19^ First order uncertainty is seen as the “classical” Bayesian idea of unknown components that we marginalize over to obtain a posterior distribution conditional on only data, while the second order uncertainty is a set of possible fixed parameters for which we want to find a best or worst case of the posterior. Characterizing second order uncertainty by a set of bias terms results in bounds on the probability representing first order uncertainty. Bounded probability is a suitable expression of uncertainty to represent expert's knowledge in situations of ignorance or ambiguity.^14^, ^15^ In the suggested method, second order uncertainty quantified within a model is seen as a difference between lower and upper bounds in quantities summarizing uncertainty about the overall effect.

The proposed framework is not limited to the RoB table. It is still valid and useful, if a different risk of bias table is used. However, modifications may be necessary particularly if there are more than three risk of bias categories. In general, we need to (i) decide the different categories of risk, (ii) assign a category of risk to each study, and (iii) specify the bounds per risk category. All steps are done on a case by case basis. In this paper, we used the RoB table because it is the recommended and therefore, most used tool to assess risk of bias in randomized clinical trials. The proposed framework makes use of the risk of bias table, so either the risk of bias table or expert's bias judgments should be available.

The suggested approach estimates quantities of interest using robust Bayesian analysis, which gives more conservative estimates of a quantity of interest compared to standard Bayesian analysis. It is, on the other hand, a more complex model to set up and usually requires the use of numerical algorithms to approximate the bounds. Bounds over the set of study qualities are in this paper approximated using a grid search approach through a discretization of the set, where Bayesian inference is done for each choice of study qualities. Additionally, an increase in the number of studies may affect the computational burden of the discretization of the elements of the set for estimating the bounds. This will be particularly so when all studies have an unclear risk of bias, in which case a larger space must be explored. An alternative approach is to search for bounds and do Bayesian updating in an iterative way using iterative importance sampling.^20^, ^21^

The choice of cut‐off values of study quality q, is subjective. That is why, our selection, (0.1,0.5,0.95), is quite conservative and includes most of the cases, as well as avoids possible numerical problems when values of q are too close to zero. The mid cut‐off value (0.5) is chosen because it is the median between 0 and 1 and links both categories of risk: high and low risk of bias. In this paper, we grouped the studies based on three different categories of risk of bias and then we specified possible values for each group. In practice, cut‐off values of study quality q can be informed from external information. For instance, future research, through meta‐epidemiological studies (analysis of multiple meta‐analysis), similar case studies in combination with expert knowledge elicitation,^5^, ^13^, ^22^ may provide empirical results to motivate less conservative choices of cut‐off values of study quality q.

An added value of robust Bayesian bias analysis, compared to a standard Bayesian bias analysis (ie, Bayesian bias analysis using a single value or a precise probability distribution of study quality) is that it can show that the conclusion is not affected by risk of bias as well as by ignorance or ambiguity regarding how to quantify the bias terms. Robust Bayesian bias analysis can be performed as a first and coarse step motivating a refined bias analysis. When adjusting for bias does (not) have a high impact on the conclusion of the meta‐analysis and therefore, a more detailed analysis may (not) be needed. A more detailed analysis could for instance gather more information regarding risk of bias or more carefully elicit bounds on study qualities. It can also be used to assess the influence of risk of bias on the conclusion of the assessment and then, if necessary, perform a Bayesian bias analysis relying on expert knowledge elicitation to specify a precise value or distribution of study quality. In the application, the bounds from adjustment made on risk of biases categorized as unclear for all studies (such as domain 1‐2) contain the bounds from any other judgment of risk of biases (Figure 3). Hence, robust Bayesian bias adjustment with unclear risk of biases is the most extreme case scenario toward a more refined approach. Robust adjustment with unclear risk of bias can be done without explicit judgments on risk of bias to explore if a quantitative bias adjustment may have a high impact on the result from the meta‐analysis. Consider, as an example, a decision maker that will approve a treatment if certainty that the overall effect is larger than t (for illustrative purposes the threshold t is set to be 1 in the application) is at least 95%. The unadjusted estimate in the application is 0.998 (Table 3), and the decision maker wants to know if adjusting for bias may have an impact on the conclusion. Robust Bayesian bias adjustment with unclear risk of bias for all studies reveals that this probability can be as low as 0.886. Since this is below 95%, it can be worthwhile to do a refined bias adjustment.

To summarize, the proposed approach provides a structured framework to consider ambiguity or ignorance in quantitative bias analysis.

## CONFLICT OF INTEREST

The authors declare no potential conflict of interests.

## AUTHOR CONTRIBUTIONS

**Ivette Raices Cruz**: Conceptualization and methodology; coding; formal analysis, visualization and writing‐original draft; writing‐review and editing. **Matthias C. M. Troffaes**: Conceptualization and methodology; writing‐review and editing. **Johan Lindström**: Conceptualization and methodology; writing‐review and editing. **Ullrika Sahlin**: Conceptualization and methodology; coding; writing‐review and editing.

## Data Availability

The JAGS and R codes to run the analysis are available at: https://github.com/Iraices/Robust_bias_adjustment.

## APPENDIX A

### A.1

The posterior expected overall effect is evaluated as:

(A1)
$$E_{q}(\mu )=\int \cdot \cdot \cdot \int \mu \cdot f_{q}(\beta_{1},\beta_{2},\beta_{3},\beta_{4},\delta_{1},\delta_{2},\delta_{3},\delta_{4},\mu ,\sigma_{\theta }^{2}|N,r,q)\;d\beta_{1}\;d\beta_{2}\;d\beta_{3}\;d\beta_{4}\;d\delta_{1}\;d\delta_{2}\;d\delta_{3}\;d\delta_{4}\;d\mu \;d\sigma_{\theta }^{2},$$

where $N=(N_{11},N_{12},N_{21},N_{22},N_{31},N_{32},N_{41},N_{42})$ and $r=(r_{11},r_{12},r_{21},r_{22},r_{31},r_{32},r_{41},r_{42})$ are the total number of patients and the number of patients who have had a positive response in control and treatment groups in each study respectively (ie, data), $q=(q_{1},q_{2},q_{3},q_{4})$ are study qualities and

(A2)
$$\begin{array}{ll} f_{q}(\beta_{1},\beta_{2},\beta_{3},\beta_{4},\delta_{1},\delta_{2},\delta_{3},\delta_{4},\mu ,\sigma_{\theta }^{2}|N,r,q) & \propto \overset{4}{\underset{i=1}{\prod }}\overset{2}{\underset{j=1}{\prod }}g^{-1}{\left(\eta_{ij}\right)}^{r_{ij}}{\left(1-g^{-1}(\eta_{ij})\right)}^{N_{ij}-r_{ij}}\cdot f(\beta_{i})\cdot f_{q_{i}}(\delta_{i}|\mu ,\sigma_{\theta }^{2})\cdot f(\mu )\cdot f(\sigma_{\theta }^{2}) \end{array}$$

is the posterior distribution for given study quality q, g(x)=logit(x), $\eta_{i1}=\beta_{i}$, $\eta_{i2}=\beta_{i}+\delta_{i}$, and $f(\beta_{i})$, $f_{q_{i}}(\delta_{i}|\mu ,\sigma_{\theta }^{2})$, f(μ) and $f(\sigma_{\theta }^{2})$ are previously specified prior distributions for parameters $\beta_{i}$, $\delta_{i}$, μ and $\sigma_{\theta }^{2}$ respectively.
